# Inflammation, Endothelial Dysfunction, and Platelet Dysregulation in Atrial Fibrillation with Chronic Kidney Disease: Toward a Biology-Informed Anticoagulation Strategy

**DOI:** 10.3390/life16040547

**Published:** 2026-03-26

**Authors:** Maria-Daniela Tanasescu, Andrei-Mihnea Rosu, Alexandru Minca, Maria-Mihaela Grigorie, Delia Timofte, Dorin Ionescu

**Affiliations:** 1 Department of Semiology-Emergency University Hospital, Carol Davila University of Medicine and Pharmacy, 022328 Bucharest, Romania; maria.tanasescu@umfcd.ro (M.-D.T.); dorin.ionescu@umfcd.ro (D.I.); 2 Department of Cardiology, Prof. Dr. Agrippa Ionescu Emergency Hospital, 077015 Balotesti, Romania; 3 Department of Dentistry, Discipline of Endodontics, Faculty of Dentistry, Carol Davila University of Medicine and Pharmacy, 020021 Bucharest, Romania; maria.grigorie@umfcd.ro; 4 Department of Dialysis, Bucharest Emergency University Hospital, 050098 Bucharest, Romania; delia.timofte@gmail.com

**Keywords:** atrial fibrillation, chronic kidney disease, inflammation, endothelial dysfunction, platelet dysfunction, anticoagulation, thrombohemorrhagic paradox, cardio–renal axis

## Abstract

Atrial fibrillation (AF) frequently coexists with chronic kidney disease (CKD), and their combination confers a disproportionate risk of both thromboembolic and bleeding events. Conventional anticoagulation strategies rely primarily on creatinine clearance-based dosing, which reflects pharmacokinetic safety but does not fully capture the biological processes underlying thrombohemorrhagic instability. This narrative review synthesizes recent mechanistic and translational evidence regarding the bidirectional cardio–renal axis in AF and CKD, focusing on systemic inflammation, endothelial dysfunction, platelet dysregulation, and altered coagulation. A structured literature search of PubMed/MEDLINE, Scopus, and Web of Science (2018–2026) was performed, complemented by manual review of key references and guidelines. The evidence indicates that inflammatory cytokine activation, oxidative stress, glycocalyx degradation, von Willebrand factor dysregulation, uremic platelet dysfunction, and enhanced thrombin generation converge to create a disrupted vascular interface in which stroke and bleeding arise from shared pathophysiological mechanisms. Renal trajectory and selected circulating biomarkers further highlight the dynamic and heterogeneous nature of risk in advanced CKD. These findings support reframing anticoagulation decision-making in AF with CKD from a static filtration-based model toward a biology-informed approach that integrates renal dynamics, endothelial and platelet phenotype, and clinical context to better align thromboembolic protection with hemorrhagic safety.

## 1. Introduction

Atrial fibrillation (AF) is the most common sustained arrhythmia worldwide, affecting an estimated 50 million individuals and contributing substantially to morbidity and mortality, largely through cardioembolic stroke [1]. Chronic kidney disease (CKD) frequently coexists with AF, and AF burden increases progressively as renal function declines. Annual AF incidence rises from 1.17 per 1000 person-years in individuals without CKD to 4.33 per 1000 person-years in stage 4 CKD, with adjusted risk estimates increasing across CKD stages and reaching 4.04 in stage 4 disease [2]. Beyond incidence alone, AF in CKD identifies a clinically vulnerable phenotype, being present in 10.6% of patients and associated with a 23% higher risk of all-cause mortality and a 45% higher risk of cardiovascular mortality [3]. This interaction may extend beyond cardiovascular outcomes, as AF has also been linked to a less favorable renal trajectory in patients with CKD. In this context, the burden becomes especially pronounced in advanced kidney disease, where AF prevalence has been estimated at 15–40% in dialysis populations [4]. At the same time, evidence to guide treatment remains limited, since only a small minority of cardiovascular randomized trials report outcomes for advanced CKD, with just 2% providing results for patients with estimated glomerular filtration rate < 30 mL/min/1.73 m^2^ and 1% for patients receiving dialysis [5]. CKD frequently coexists with AF and adds considerable clinical complexity. Thromboembolic risk scores used in routine practice demonstrate only modest predictive performance in patients with renal impairment and do not fully capture CKD-related determinants of stroke risk, whereas bleeding risk models explicitly incorporate renal dysfunction and dialysis status [1]. This therapeutic dilemma is especially pronounced in advanced kidney failure (estimated glomerular filtration rate < 15 mL/min/1.73 m^2^), where patients with AF remain at high risk of both thromboembolic and bleeding events, while major randomized trials of oral anticoagulants have largely excluded those with severely reduced kidney function [6]. Consequently, the net clinical benefit of anticoagulation in end-stage renal disease remains uncertain, and alternative strategies such as left atrial appendage occlusion are increasingly considered in carefully selected high-risk individuals [7].

CKD is characterized by the coexistence of thrombotic and hemorrhagic tendencies. Progressive renal dysfunction is associated with chronic inflammation, increased fibrinogen and factor VIII levels, elevated von Willebrand factor (Vwf), reduced endogenous anticoagulant activity, and activation of the renin–angiotensin–aldosterone system, which promotes hypofibrinolysis [8]. Uremia also alters platelet behavior. Enhanced *in vivo* platelet activation, increased P-selectin expression, αIIbβ3 activation, and circulating procoagulant microparticles favor thrombin generation. At the same time, platelet adhesion and aggregation may be impaired. Abnormal interaction with Vwf and fibrinogen, storage pool defects, reduced thromboxane A_2_ production, nitric oxide-mediated inhibition, anemia, and dialysis-related factors contribute to bleeding susceptibility [8]. In anticoagulated AF populations, systemic inflammatory states have been linked to simultaneous increases in thrombotic events and bleeding complications, suggesting that inflammation may amplify both processes rather than shift hemostasis in a single direction [9].

AF itself is increasingly viewed as a systemic inflammatory and endothelial disorder rather than solely an electrical disturbance. Circulating inflammatory mediators, including interleukin-6, tumor necrosis factor-α, C-reactive protein, and cell-free mitochondrial DNA, are consistently elevated [10,11]. Experimental evidence connects mitochondrial dysfunction, reactive oxygen species (ROS) generation, and redox-sensitive signaling pathways to atrial remodeling and electrical instability [11]. Endothelial perturbation further contributes to this prothrombotic milieu. Disturbed shear stress during AF is associated with increased expression of adhesion molecules, tissue factor exposure, enhanced Vwf release, and altered nitric oxide (NO) bioavailability [10]. Hematologic indices such as the neutrophil-to-lymphocyte ratio, platelet-to-lymphocyte ratio, monocyte-to-lymphocyte ratio, and red cell distribution width correlate with AF occurrence and recurrence, reflecting sustained inflammatory and platelet activation states [12].

Despite these advances in biological understanding, stroke prevention strategies in AF remain largely grounded in static clinical scores and creatinine clearance (CrCl)-based estimates of renal function. Even modified schemes incorporating renal impairment show limited discrimination, particularly in dialysis populations [1,6]. Standard coagulation assays provide limited insight into the complex and dynamic hemostatic disturbances that accompany kidney failure [13]. The simultaneous rise in thrombotic and bleeding events in patients with AF and CKD therefore challenges risk stratification models that rely mainly on static clinical variables and creatinine-based estimates of renal function.

This review explores how inflammation, endothelial dysfunction, and platelet dysregulation interact across the cardio–renal axis to shape thrombohemorrhagic risk in patients with AF and CKD. Particular attention is given to distinguishing mechanisms that favor thrombosis, mechanisms that promote bleeding susceptibility, and the areas of biological overlap through which these processes coexist. By integrating mechanistic and translational evidence, we propose a biologically informed framework that may help refine anticoagulation decision-making beyond creatinine-based assessment.

## 2. Materials and Methods

We sought to evaluate and synthesize current evidence regarding the inflammatory, endothelial, and platelet-mediated mechanisms underlying the paradoxical coexistence of thrombotic propensity and bleeding susceptibility in patients with AF and CKD. Specifically, we examined (i) the bidirectional pathophysiological interactions between AF and CKD; (ii) the contribution of systemic inflammation and oxidative stress to endothelial dysfunction; (iii) platelet activation and uremic platelet dysfunction; and (iv) the implications of these biological pathways for anticoagulation strategies and risk stratification beyond CrCl-based models.

A comprehensive literature search was conducted in PubMed/MEDLINE, Scopus, and Web of Science from January 2018 through January 2026. The search strategy combined Medical Subject Headings (MeSH) and free-text keywords, including: (“atrial fibrillation” OR “AF”) AND (“chronic kidney disease” OR “CKD” OR “renal dysfunction” OR “uremia”) AND (“inflammation” OR “oxidative stress” OR “cytokines” OR “NLRP3”) AND (“endothelial dysfunction” OR “nitric oxide” OR “von Willebrand factor” OR “microvascular injury”) AND (“platelet activation” OR “platelet dysfunction” OR “hemostasis” OR “thrombosis” OR “bleeding”) AND (“anticoagulation” OR “direct oral anticoagulants” OR “warfarin”).

Additional relevant studies were identified through manual screening of reference lists from key primary publications, recent systematic reviews, consensus statements, and major cardiology and nephrology guidelines.

Eligible sources included: (a) original research articles encompassing observational studies, randomized controlled trials, translational investigations, and mechanistic experimental studies; (b) systematic reviews and meta-analyses; (c) authoritative narrative reviews providing mechanistic or conceptual frameworks; and (d) relevant clinical and consensus guidelines. Articles published in English were prioritized. Case reports, editorials, and conference abstracts were excluded unless they provided unique mechanistic insights not otherwise represented in the peer-reviewed literature.

Given the narrative nature of this review, no formal risk-of-bias assessment or quantitative quality scoring was performed. Instead, study inclusion prioritized mechanistic depth, biological plausibility, translational relevance, and conceptual contribution to understanding the thrombohemorrhagic paradox in AF with CKD. Particular emphasis was placed on studies evaluating biological markers beyond CrCl.

## 3. The Cardio–Renal Axis in Atrial Fibrillation and Chronic Kidney Disease

### 3.1. Atrial Fibrillation as a Driver of Renal Dysfunction

AF is increasingly recognized as a condition associated with progressive renal impairment. In community-based cohorts, newly diagnosed AF is linked to a higher risk of incident CKD and lower estimated glomerular filtration rate compared with individuals without AF [14]. Within established AF populations, the coexistence of heart failure and CKD identifies patients at particularly high risk, with rates of renal death and dialysis rising stepwise across worsening of the estimated glomerular filtration rate (eGFR) categories [15]. Similar findings have been reported in elderly cohorts, where AF in the setting of heart failure and renal dysfunction is associated with accelerated progression toward advanced kidney disease and adverse cardiorenal outcomes [16]. These data suggest that AF is not merely a bystander but may contribute to ongoing renal decline, especially when ventricular dysfunction is present.

Hemodynamic disturbance may represent one contributing mechanism. In patients with AF, particularly those with concomitant heart failure or impaired ventricular function, altered cardiac performance may favor progressive renal impairment. In AF populations, the coexistence of heart failure and CKD is associated with higher rates of renal death and dialysis [15], and large-scale population analyses confirm that combined cardiac and renal dysfunction defines a phenotype at high risk for advanced renal outcomes [16]. Neurohormonal activation likely contributes further to this process. Activation of the renin–angiotensin–aldosterone system promotes sodium retention, volume expansion, and renal vascular stress, thereby accelerating structural and functional kidney injury [17].

AF is also associated with sympathetic activation and persistent stimulation of the renin–angiotensin–aldosterone system (RAAS), leading to vasoconstriction, oxidative stress, and profibrotic signaling in both cardiac and renal tissue [18]. In patients with AF and CKD, worsening renal function correlates with adverse cardiovascular outcomes, emphasizing the reciprocal relationship between declining kidney function and atrial remodeling [19]. This interaction extends beyond short-term hemodynamic compromise and reflects ongoing structural remodeling across both organs.

Markers of renal microvascular injury further support a shared pathophysiology. Even low-grade albuminuria in AF is associated with higher risks of heart failure, stroke, bleeding, and mortality, independent of traditional risk factors [20]. Community data demonstrate a graded increase in cardiovascular risk across declining eGFR and rising urinary albumin-to-creatinine ratio (UACR) categories among patients with AF [14]. Metabolic factors also contribute. Reduced kidney function and elevated uric acid levels are associated with incident AF, indicating overlapping inflammatory and metabolic pathways [21]. In patients with AF and established CKD, progressive renal impairment frequently clusters with both ischemic and bleeding complications, reinforcing the contribution of endothelial and inflammatory dysregulation to cardiorenal progression [22].

In clinical practice, these observations emphasize that renal decline in AF is rarely incidental. It reflects the interaction of congestion, neurohormonal activation, endothelial injury, and systemic inflammation that evolves over time and influences both cardiovascular and renal prognosis.

### 3.2. CKD as a Pro-Arrhythmic and Pro-Thrombotic State

As kidney function declines, a wide range of uremic retention molecules accumulates in the circulation, including protein-bound solutes such as indoxyl sulfate and p-cresyl sulfate [23]. These compounds exert biological effects through receptor-mediated pathways and sustained cellular stress, contributing to oxidative injury, endothelial dysfunction, and altered interorgan signaling. In patients with CKD, accumulation of uremic toxins is associated with myocardial hypertrophy, interstitial fibrosis, and electrical remodeling—features characteristic of uremic cardiomyopathy and closely linked to atrial arrhythmias [24]. Gut-derived metabolites such as trimethylamine-N-oxide further enhance oxidative stress and activate inflammatory and profibrotic pathways within cardiac tissue, facilitating structural remodeling and electrical instability [25]. The uremic milieu therefore promotes conditions favorable to atrial arrhythmogenesis through metabolic and inflammatory mechanisms that extend beyond simple volume overload.

Oxidative stress represents a central mechanism in this process. In the setting of CKD, sustained oxidative injury and metabolic stress may impair mitochondrial function, thereby increasing ROS production, disrupting adenosine triphosphate (ATP) homeostasis, and altering calcium handling. Excess ROS can promote ryanodine receptor 2 (RyR2) dysregulation and reduce sarcoplasmic reticulum Ca^2+^-ATPase (SERCA) activity, leading to intracellular calcium overload and creating conditions favorable to triggered activity and re-entrant circuits. Oxidative stress may also modify ion channels and impair gap junction integrity through connexin dysregulation, thereby increasing conduction heterogeneity and electrical dispersion across the atrial myocardium [11]. These changes provide a mechanistic link between systemic oxidative stress and atrial electrical instability.

Vascular and endothelial alterations further reinforce this arrhythmogenic substrate. CKD is associated with mineral metabolism disturbances that promote vascular smooth muscle cell osteogenic transition, medial calcification, and arterial stiffening [26]. Hyperphosphatemia, elevated fibroblast growth factor 23, reduced Klotho expression, and diminished levels of calcification inhibitors such as matrix Gla protein and fetuin-A contribute to progressive vascular rigidity and endothelial dysfunction [26]. Endothelial injury, characterized by reduced NO bioavailability and increased oxidative stress, impairs microvascular perfusion and favors atrial fibrotic remodeling. These structural and microvascular changes alter atrial conduction properties and lower the threshold for sustained arrhythmia [27,28].

CKD also promotes a pro-thrombotic vascular phenotype. Uremic toxins such as indoxyl sulfate activate inflammatory signaling pathways and enhance tissue factor expression in endothelial and vascular cells [24,25]. Persistent oxidative stress and inflammasome activation support a pro-coagulant state at the vascular interface [26]. Lipid-derived intermediates, including ceramides and oxidized low-density lipoprotein particles, impair mitochondrial energetics and amplify interstitial fibrosis while enhancing platelet reactivity within the cardiovascular–kidney–metabolic axis [29]. These processes contribute to endothelial activation, platelet priming, and altered fibrinolysis.

**Figure 1:**
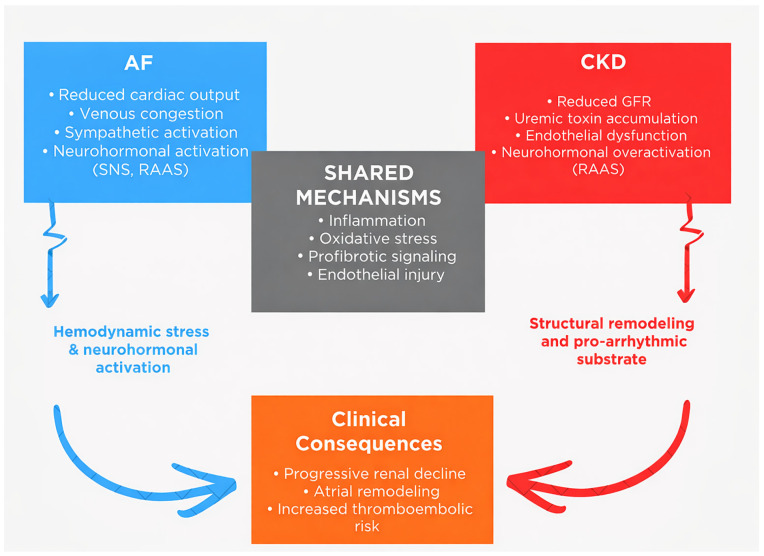
Bidirectional cardio–renal axis in atrial fibrillation and chronic kidney disease.

From a clinical perspective, CKD therefore acts as a biologically active driver of both arrhythmia and thrombosis. Structural remodeling, oxidative injury, endothelial dysfunction, and metabolic disturbance converge to create a myocardial and vascular environment susceptible to atrial fibrillation and embolic complications. When AF develops in this setting, it does so on a substrate already predisposed to electrical instability and pro-coagulant activity, helping to explain the heightened thromboembolic burden observed in patients with concurrent CKD [24,29] (Figure 1).

## 4. Systemic Inflammation as a Central Amplifier

### 4.1. Cytokine Activation and Oxidative Stress

Systemic inflammation is a central component of the pathophysiological link between AF and CKD. As renal function declines, persistent activation of innate immune pathways leads to sustained cytokine release and oxidative stress, promoting endothelial injury, myocardial remodeling, and a prothrombotic phenotype.

In CKD, the accumulation of uremic toxins and immune dysregulation drives chronic low-grade inflammation. Circulating interleukin-6 (IL-6), tumor necrosis factor-α (TNF-α), and C-reactive protein (CRP) rise progressively with worsening kidney function. These mediators actively contribute to endothelial activation, vascular injury, and accelerated atherosclerosis. Coronary plaques in CKD demonstrate greater inflammatory cell infiltration than those in individuals without renal impairment, supporting a mechanistic association between inflammatory burden and vascular disease [30].

Within this inflammatory network, IL-6 enhances hepatic CRP synthesis and amplifies endothelial dysfunction, whereas TNF-α upregulates adhesion molecule expression and augments oxidative stress, facilitating leukocyte recruitment and microvascular damage. This cytokine-driven environment not only sustains vascular injury but also promotes myocardial remodeling. A further layer of amplification is provided by the NLRP3 inflammasome, which links metabolic stress and oxidative signals to cardiovascular instability. Upon activation, NLRP3 induces caspase-1–dependent maturation of IL-1β and IL-18, intensifying downstream cytokine cascades, including IL-6 production [31]. Enhanced NLRP3 signaling has been associated with plaque destabilization and adverse myocardial remodeling, positioning inflammasome activity at the interface of thrombosis and arrhythmogenesis [31].

Experimental data support a direct electrophysiological impact of inflammasome activation. In a murine model of diabetes-associated AF, atrial macrophage infiltration increased IL-1β expression and mitochondrial reactive oxygen species (mitoROS) production; both IL-1β neutralization and macrophage depletion reduced AF inducibility [32]. These observations indicate that IL-1β-mediated inflammation can alter atrial electrical properties even in the absence of advanced fibrosis.

Oxidative stress represents the principal downstream effector of this inflammatory cascade. Cytokine signaling impairs mitochondrial function, activates NADPH oxidases, and diminishes antioxidant capacity, leading to excess ROS generation. In diabetic atria, elevated mitoROS levels correlated with arrhythmia susceptibility, and mitochondria-targeted antioxidants attenuated AF inducibility [32]. At the cellular level, ROS modify redox-sensitive pathways involved in calcium handling. Oxidative activation of Ca^2+^/calmodulin-dependent protein kinase II (CaMKII) increases ryanodine receptor 2 (RyR2) phosphorylation and sarcoplasmic reticulum Ca^2+^ leak, promoting triggered activity and electrical instability [32].

In CKD, sustained cytokine activation and oxidative stress converge to create a pro-oxidative and prothrombotic milieu [30,31]. This inflammatory–oxidative axis accelerates vascular aging, destabilizes atherosclerotic plaques, and disrupts atrial calcium homeostasis. Through these interconnected mechanisms, systemic inflammation contributes directly to atrial arrhythmogenesis and the heightened thrombotic risk observed in patients with AF and CKD.

### 4.2. Inflammation–Coagulation Cross-Talk

Inflammation and coagulation are biologically interdependent systems. Contemporary models of hemostasis extend beyond the classical enzymatic cascade to incorporate innate immune activation as a central regulator of thrombosis [33]. This integrated framework, often termed thromboinflammation or immunothrombosis, describes coagulation as a cellular process embedded within immune signaling networks [34].

Activated platelets, tissue factor expression, thrombin generation, and innate immune effectors such as neutrophil extracellular traps (NETs) form the core of this interface. Platelet-derived extracellular vesicles provide procoagulant surfaces that enhance thrombin generation, while inflammatory and hypoxic signaling promote tissue factor expression on monocytes and endothelial cells [35,36]. Innate immune activation further reinforces this shift through Toll-like receptor signaling, which promotes cytokine release, endothelial activation, and additional tissue factor expression [37]. Thrombin amplifies this response through feedback activation of coagulation and protease-activated receptor signaling, thereby linking clot formation to endothelial activation and vascular inflammation [27,28,30]. NETs strengthen this procoagulant environment by supporting platelet adhesion, factor XII activation, and additional thrombin generation [38].

In AF and CKD, these pathways intersect. AF-associated flow disturbance favors platelet activation, while CKD is characterized by persistent inflammatory signaling and enhanced circulating procoagulant vesicles [35,36,37,38]. The coexistence of these conditions lowers the threshold for thrombin generation and sustains feed-forward interactions between immune activation and coagulation, contributing to the thromboinflammatory state that promotes clot formation while also predisposing to hemostatic instability—Table 1.

## 5. Endothelial Dysfunction and Microvascular Injury

### 5.1. Nitric Oxide Imbalance and eNOS Dysfunction

Reduced NO bioavailability is a defining feature of endothelial dysfunction in CKD and assumes particular importance in patients with AF, in whom endothelial instability contributes to both thrombosis and microvascular injury. In CKD, impaired NO signaling reflects diminished synthesis and increased degradation. Circulating levels of asymmetric and symmetric dimethylarginine (ADMA and SDMA), endogenous inhibitors of endothelial nitric oxide synthase (eNOS), rise as renal function declines and directly suppress NO production. Chronic inflammation and oxidative stress further attenuate eNOS activity, while CKD-specific disturbances, such as hyperphosphatemia, elevated fibroblast growth factor 23, and reduced Klotho expression, exacerbate endothelial dysfunction through NO-dependent mechanisms [39].

Uremic retention solutes provide an additional insult. Indoxyl sulfate, p-cresyl sulfate, and inorganic phosphate impair endothelium-dependent vasorelaxation, reduce NO bioavailability, and stimulate ROS generation. Increased oxidative stress accelerates NO consumption and promotes further accumulation of ADMA, creating a cycle in which endothelial injury perpetuates its own progression [40].

AF compounds this dysfunction. Loss of coordinated atrial contraction alters shear stress patterns that normally maintain eNOS expression and phosphorylation. Both clinical and experimental data demonstrate reduced eNOS expression and diminished NO levels in AF, particularly within the left atrial appendage, accompanied by increased plasminogen activator inhibitor-1 expression—changes that favor local thrombogenesis [41]. Thus, AF imposes hemodynamic and molecular stress on an already compromised vascular substrate in CKD.

Oxidative stress can shift eNOS toward a dysfunctional state in which superoxide generation increases at the expense of NO production. Oxidation of tetrahydrobiopterin (BH4), a reduced BH4/BH2 ratio, substrate limitation, and ADMA excess favor this transition [41]. Superoxide rapidly reacts with NO to form peroxynitrite, further oxidizing BH4 and amplifying nitroxidative stress. The result is sustained endothelial dysfunction characterized by impaired vasodilatory reserve, heightened thrombotic tendency, and microvascular fragility in patients with concurrent AF and CKD [40,41,42].

### 5.2. Glycocalyx Degradation

The endothelial glycocalyx is a highly organized, polysaccharide-rich layer that lines the luminal surface of the vascular endothelium. It is composed of membrane-bound proteoglycans, including syndecans and glypicans, glycosaminoglycans such as heparan sulfate and hyaluronan, and adsorbed plasma proteins that together create a negatively charged interface regulating permeability, leukocyte trafficking, and hemostatic balance [37,38]. In CKD, progressive glycocalyx injury is supported by consistent biochemical evidence. Circulating markers of shedding, particularly syndecan-1 and hyaluronan, are elevated in CKD and reach their highest levels in dialysis-dependent patients. These elevations parallel increases in vWF and vascular cell adhesion molecule-1, reflecting concomitant endothelial activation [43]. Importantly, glycocalyx degradation markers correlate with concentrations of indoxyl sulfate and p-cresyl sulfate, linking the uremic milieu directly to structural disruption of the endothelial surface layer [43].

Under physiological conditions, the glycocalyx is continuously renewed, maintaining equilibrium between synthesis and enzymatic degradation [44]. Heparan sulfate chains play a central role in mechanotransduction. Their integrity is required for shear stress-mediated activation of eNOS; experimental removal of heparan sulfate abolishes flow-induced NO production, underscoring its importance in vascular homeostasis [44]. Loss of this structural framework therefore compromises both barrier function and vasodilatory signaling.

Oxidative stress and inflammation drive glycocalyx degradation in CKD., ROS, pro-inflammatory cytokines, ischemia–reperfusion injury, catecholamines, and activation of heparanases and matrix metalloproteinases promote shedding of glycocalyx components. The resulting denudation increases vascular permeability and facilitates leukocyte and platelet adhesion, contributing to endothelial activation and microvascular dysfunction [45]. In CKD, these structural alterations occur in parallel with biochemical markers of endothelial injury and uremic toxin burden, supporting a mechanistic link between the uremic environment and glycocalyx destabilization [43].

### 5.3. Von Willebrand Factor Alterations

vWF is synthesized by endothelial cells and stored in Weibel–Palade bodies, from which it is released following endothelial activation or injury. In AF, biochemical evidence of endothelial dysfunction is detectable even in the absence of overt structural heart disease, and circulating vWF concentrations are consistently elevated. Loss of coordinated atrial contraction and the development of turbulent flow reduce laminar shear stress, impair endothelial eNOS activity, and diminish NO bioavailability. These hemodynamic perturbations favor endothelial activation and promote vWF release into the circulation [41].

CKD amplifies this endothelial imbalance. In a large prospective cohort of dialysis patients, elevated vWF levels were independently associated with increased mortality. The association was strongest in individuals with reduced ADAMTS13 activity [46]. As ADAMTS13 cleaves ultra-large vWF multimers into smaller, less thrombogenic forms, decreased enzymatic activity permits persistence of highly adhesive multimers that enhance platelet tethering and aggregation under high shear conditions.

Endothelial biomarker profiling in paroxysmal AF demonstrates measurable vWF levels alongside soluble adhesion molecules and thrombomodulin, indicating sustained endothelial activation even in clinically stable populations [47]. Disturbed shear stress in AF further alters endothelial phenotype and attenuates signaling pathways that preserve antithrombotic surface properties [48].

In the combined setting of AF and CKD, enhanced vWF release and impaired multimer processing create a vascular surface predisposed to platelet adhesion and thrombus formation. The imbalance between vWF secretion and ADAMTS13-mediated regulation provides a mechanistic link between endothelial dysfunction and the increased thromboembolic risk observed in this high-risk population [46,47,48].

### 5.4. Endothelial–Platelet Interaction

Under physiological conditions, the intact endothelium maintains platelets in a quiescent state. A negatively charged glycocalyx limits platelet contact with adhesion molecules, while endothelial-derived NO and prostacyclin inhibit platelet activation through cyclic nucleotide signaling and reduction in intracellular calcium. Endothelial CD39 further attenuates platelet reactivity by hydrolyzing ATP and ADP, reducing amplification of aggregation responses [49].

Once platelet activation occurs, granule secretion and receptor upregulation strengthen interactions with the vessel wall. α-Granules contain adhesive and coagulation-related proteins including Vwf and fibrinogen, and activation increases surface expression of key receptors that support adhesion and aggregation. Platelet P-selectin mediates interactions with endothelial cells and leukocytes, while integrin αIIbβ3 binds plasma ligands such as fibrinogen and Vwf, stabilizing platelet–cell and platelet–platelet adhesion [50].

Hemodynamic conditions shape these processes. In flow models, platelet adhesion to vascular surfaces depends strongly on shear rate, with Vwf acting as a key intermediate that promotes platelet deposition at higher shear. Platelet activation and aggregation then couple to coagulation: tissue factor-driven thrombin generation activates platelets via protease-activated receptors, and highly activated platelets provide a procoagulant phospholipid surface that accelerates thrombin generation and supports fibrin formation [51].

Endothelial cells can also actively suppress platelet adhesion and activation under flow. Using high-shear whole-blood microfluidics with endothelial monolayers, the presence of human endothelial cells markedly reduced platelet adhesion and activation. This suppression involved modulation of platelet signaling downstream of GPVI- and PAR-mediated activation pathways, and phosphoproteomic analyses indicated broad resetting of platelet phosphorylation networks that extended beyond classical NO and prostacyclin effects [52].

**Figure 2:**
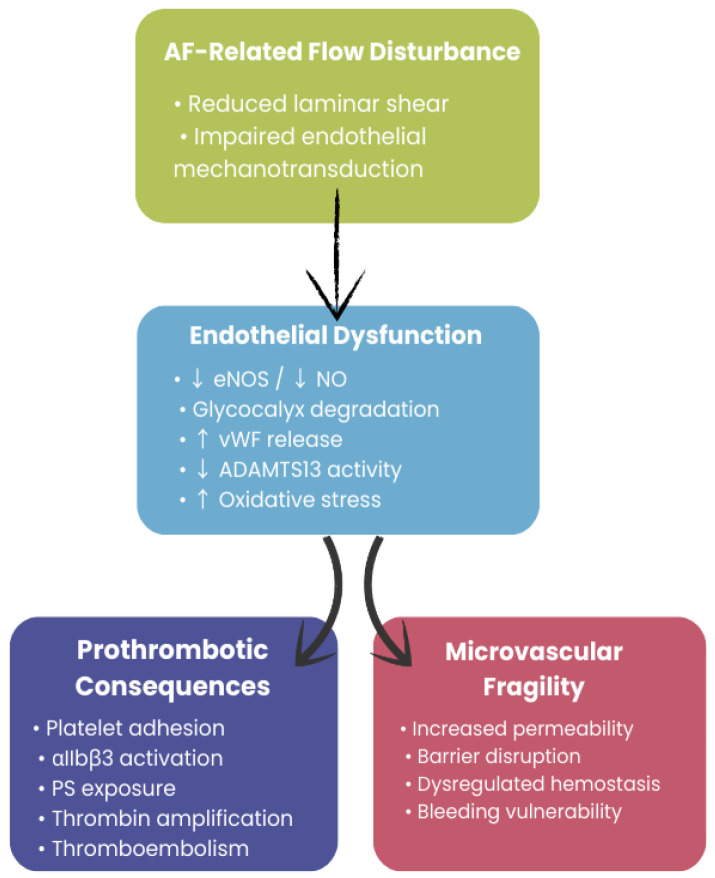
Endothelial dysfunction as a convergent mechanism linking atrial fibrillation–related flow disturbance to prothrombotic and microvascular consequences in CKD. Downward arrows (↓) indicate reduced biological activity or availability. Upward arrows (↑) indicate increased expression or activity.

The convergence of endothelial injury and platelet activation, which may help explain the coexistence of prothrombotic signaling and bleeding susceptibility, is schematically illustrated in Figure 2.

## 6. Platelet Dysregulation in CKD and AF

### 6.1. Pro-Thrombotic Platelet Activation

Patients with CKD exhibit a persistent state of *in vivo* platelet activation that contributes to heightened thrombotic risk. Circulating platelets in CKD demonstrate increased surface expression of P-selectin and enhanced formation of platelet–leukocyte aggregates, reflecting a primed phenotype even in the absence of overt vascular injury [13]. Uremia, oxidative stress, inflammation, and dialysis-related hemoincompatibility further amplify platelet activation, promoting exposure of procoagulant phosphatidylserine and release of platelet-derived extracellular vesicles (pEVs) [13]. These alterations favor thrombin generation and contribute to a hypercoagulable milieu.

P-selectin exposure on activated platelets has functional consequences beyond serving as a biomarker. It mediates adhesion to leukocytes and endothelial cells, facilitating thromboinflammatory interactions that sustain local coagulation and vascular injury [34]. In inflammatory settings, dysregulated activation of platelets and coagulation factors promotes reciprocal amplification, with platelet activation enhancing tissue factor–dependent pathways and thrombin generation, while thrombin further activates platelets via protease-activated receptors [34,51]. This bidirectional signaling lowers the threshold for thrombus formation in susceptible vascular territories.

Shear stress critically regulates platelet adhesion and aggregation. Under high wall-shear rates, platelet adhesion becomes strongly dependent on vWF, which tethers platelets through GPIb-IX-V and stabilizes adhesion via integrin αIIbβ3 [51]. Experimental flow chamber models demonstrate that increasing shear enhances platelet deposition on collagen- and vWF-coated surfaces, triggering activation, aggregation, and procoagulant activity [51]. Shear-dependent platelet aggregation has been linked to arterial thrombotic disorders characterized by disturbed flow conditions [51]. In AF, where blood flow patterns are altered and regional shear gradients may vary, these mechanisms may plausibly contribute to thrombus formation.

Activated platelets also release pEVs enriched in phosphatidylserine, tissue factor, factor Va, and factor VIII, providing catalytic surfaces that accelerate assembly of tenase and prothrombinase complexes [35]. These vesicles disseminate procoagulant activity beyond the initial site of activation and reinforce thrombin generation. pEVs further express adhesion molecules, including CD62P (P-selectin), facilitating cellular cross-talk within the thrombus microenvironment [35].

In CKD and AF, sustained platelet activation, shear-dependent adhesion, and vesicle-mediated amplification converge to promote thrombin generation and intensified platelet–leukocyte–endothelial interactions [13,34,35,51].

### 6.2. Uremic Platelet Dysfunction

Clinical assessment in CKD consistently indicates impaired primary hemostasis, frequently manifested by prolonged bleeding time and reduced platelet adhesion. Evidence synthesis focusing on nondialysis-related CKD shows that platelet adhesion is commonly diminished and maximal ex vivo aggregation, particularly in response to collagen, is reduced, although results vary according to agonist and assay methodology [53]. This variability reflects the heterogeneous laboratory phenotype of uremic thrombocytopathy, in which platelet counts may remain near normal while functional performance at sites of vascular injury is compromised.

A mechanistic link between uremia and defective aggregation involves post-translational modification of platelet proteins by urea-derived cyanate. In patients receiving hemodialysis, substantial carbamylation of the αIIbβ3 integrin has been demonstrated, accompanied by reduced activation-dependent PAC-1 binding and impaired fibrinogen binding, together with slower and attenuated aggregation responses. Experimental cyanate exposure showed impaired adhesion to fibrinogen and altered thrombin-induced aggregation kinetics. Site-directed mutagenesis identified lysine 185 within the β3 subunit as critical for receptor activation; loss of positive charge at this residue prevents the conformational transition required for high-affinity fibrinogen binding, thereby functionally resembling an acquired integrin defect. Importantly, PAR-1 cleavage remains preserved, and translocation of α-granule markers such as P-selectin and CD40L is unaffected, indicating that impaired aggregation primarily reflects defective integrin activation rather than generalized signaling or secretion failure [54].

Adhesion abnormalities in CKD also involve alterations in Vwf. Carbamylation modifies vWF interactions with its binding partners, including reduced binding to collagen types I and III and impaired interaction with platelets, changes that would be expected to weaken platelet tethering at sites of endothelial disruption. Concurrently, platelet responses demonstrate a complex pattern: thrombin-dependent activation is associated with increased phosphatidylserine exposure, whereas thromboxane A_2_ release is reduced. This profile suggests that enhanced procoagulant surface generation may coexist with impaired signaling pathways required for stable hemostatic plug formation [55].

In the hemodialysis setting, intrinsic uremic dysfunction is further influenced by circuit-related platelet activation. Increased CD62P expression across the dialysis circuit, enhanced platelet–leukocyte aggregation, progressive reduction in platelet size during treatment, and ultrastructural evidence of reduced dense granule content have been described [56]. These features are consistent with partial degranulation and a state of platelet exhaustion, potentially limiting the capacity to mount an effective hemostatic response when vascular injury occurs.

### 6.3. Determinants of Hemorrhagic Risk

Bleeding risk in patients with CKD and AF is influenced by systemic and dialysis-related factors that directly affect primary hemostasis. Among these, anemia has a measurable impact on platelet function. Red blood cell (RBC) mass determines blood viscosity and intraluminal flow patterns, which regulate platelet margination. Under physiological conditions, RBCs occupy the axial stream and displace platelets toward the endothelial surface, facilitating rapid recruitment at sites of vascular injury. When hematocrit decline, particularly below ~30%, this shear-dependent redistribution is reduced, leading to impaired platelet–wall interactions and weaker adhesion under flow [57].

Blood rheology further modifies this effect. As a non-Newtonian fluid, blood exhibits viscosity changes that depend on hematocrit and shear rate. Reduced RBC mass alters this relationship and diminishes the mechanical forces required for efficient platelet tethering and aggregation in arterial and microvascular beds. Lower hematocrit therefore compromises early hemostatic plug formation, especially under high-shear conditions. RBCs also contribute to clot formation and stability. They provide a surface that supports thrombin generation and influence fibrin architecture. Decreased RBC availability may limit local thrombin amplification and produce less compact thrombi with reduced mechanical resilience [58,59]. Clinical data are consistent with these mechanisms: lower hemoglobin levels are associated with impaired primary hemostasis and higher bleeding rates. In anticoagulated patients, baseline anemia predicts major bleeding and mortality, emphasizing its relevance as a modifiable determinant of hemorrhagic risk [58].

Hemodialysis introduces additional perturbations. Exposure of blood to artificial membranes results in rapid protein adsorption, conformational changes, and activation of coagulation and inflammatory pathways. Contact activation promotes intrinsic pathway signaling, thrombin generation, platelet adhesion, and complement activation [60,61]. Routine heparinization attenuates thrombin formation but does not suppress complement activation or platelet–leukocyte interactions induced by membrane contact [61]. Subclinical coagulation and inflammatory activation therefore persist during dialysis sessions.

Repeated intradialytic stimulation promotes platelet adhesion to fibrinogen-coated surfaces via αIIbβ3, exposure of procoagulant phospholipids, and formation of platelet–fibrin aggregates within the extracorporeal circuit [61]. Membrane composition significantly influences the magnitude of these responses. Hydrophobic materials and surfaces with high protein adsorption capacity are associated with greater complement activation and procoagulant signaling, whereas hydrophilic or surface-modified membranes attenuate protein binding and subsequent cellular activation [60,61].

Complement activation peaks early after dialysis initiation and does not fully normalize by session end, reflecting ongoing bioincompatibility-related stimulation [62]. Anaphylatoxins such as C3a and C5a promote leukocyte activation, oxidative stress, and cross-talk with coagulation pathways, reinforcing thrombin generation and platelet activation [61,62]. Although dialysis removes certain circulating inflammatory mediators, it simultaneously induces complement activation through membrane contact and extracorporeal circulation [62]. Recurrent exposure to this stimulus, superimposed on the inflammatory milieu of advanced CKD, contributes to chronic endothelial dysfunction.

Over time, repeated contact activation and platelet consumption may result in functional platelet exhaustion, compounding intrinsic uremic platelet defects. Dialysis modality modifies this response: convective techniques and highly biocompatible membranes are associated with lower inflammatory activation compared with conventional high-flux hemodialysis [62]. Dialysis-related bleeding risk therefore reflects a dynamic balance between intradialytic platelet activation and progressive platelet dysfunction or consumption. Membrane characteristics, complement activation kinetics, anticoagulation strategy, and treatment modality should be considered when evaluating bleeding susceptibility in patients undergoing chronic hemodialysis.

Hemodialysis is also associated with sustained perturbation of the vWF–ADAMTS13 axis. In a prospective dialysis cohort, vWF levels were markedly elevated, whereas ADAMTS13 activity was reduced compared with reference populations. Patients in the highest vWF quartile had a 1.4-fold increase in all-cause mortality, and those in the lowest ADAMTS13 quartile had a 1.3-fold increase. Concomitant high vWF and low ADAMTS13 activity doubled mortality risk, affecting both cardiovascular and non-cardiovascular outcomes [46]. These findings reflect ongoing endothelial activation and impaired cleavage of ultra-large vWF multimers.

Elevated vWF levels do not confer protection against bleeding. In hemodialysis-dependent ESRD, both vWF antigen and ristocetin cofactor activity (vWF:RCo) are significantly increased compared with controls and rise as renal function declines. Among dialysis patients with gastrointestinal bleeding, vWF:RCo values are higher than in those without bleeding and remain independently associated with hemorrhagic events in multivariable analysis [63]. Receiver operating characteristic analyses demonstrate high sensitivity of both vWF-Ag and vWF:RCo for identifying bleeding risk.

The coexistence of elevated vWF activity and increased bleeding suggests qualitative dysregulation rather than simple quantitative deficiency. Excess endothelial release of vWF combined with reduced ADAMTS13 activity alters multimer composition and functional balance. These alterations create a hemostatic profile marked by enhanced shear-dependent platelet adhesion, sustained endothelial activation, and abnormal thrombus architecture. This configuration may promote thrombotic events while increasing vulnerability to clinically significant bleeding—Table 2.

## 7. The Thrombohemorrhagic Paradox in AF with CKD

AF remains a major cause of ischemic stroke, and long-term oral anticoagulation is recommended in patients with elevated thromboembolic risk [58]. When CKD coexists, however, the clinical landscape changes substantially. As renal function declines, both thromboembolic and major bleeding events increase, particularly in advanced CKD and ESRD [64,65,66]. In dialysis populations, absolute event rates are high, and uncertainty persists regarding the net clinical benefit of anticoagulation [65,66].

What distinguishes AF with CKD from many other high-risk states is that stroke and bleeding do not move in opposite directions—they rise in parallel [64,65,66]. Conventional clinical tools, including CHA_2_DS_2_-VASc and bleeding risk scores, provide a necessary framework for decision-making but lose discriminatory performance in advanced renal impairment. Attempts to enhance stroke prediction by incorporating renal function into risk models have produced inconsistent improvements [64]. These observations suggest that AF with CKD reflects a shared biological substrate rather than two competing and independent risks.

Endothelial activation and altered vWF regulation sit at the center of this convergence. Inflammatory stimuli trigger vWF release from endothelial Weibel–Palade bodies, increasing circulating concentrations and promoting formation of hyperadhesive multimers. Under shear stress, vWF unfolds, exposing platelet-binding domains and the ADAMTS13 cleavage site. When cleavage is reduced or functionally impaired, large multimers persist and enhance platelet tethering and aggregation [67]. Physiologic hemostasis becomes maladaptive when this axis is amplified. CKD reinforces this pathway at several levels. Uremic serum promotes endothelial activation, increasing vWF and tissue factor expression in parallel with oxidative and inflammatory signaling [68]. ROScan modify vWF conformation and reduce susceptibility to ADAMTS13-mediated cleavage, stabilizing a more adhesive phenotype [61]. Endothelial cells in CKD also demonstrate enhanced adhesion molecule expression and a prothrombotic extracellular matrix [68]. Superimposed AF-related flow disturbance (atrial dilation, remodeling, and regional stasis) creates local conditions favoring vWF self-association and platelet recruitment [67]. CKD therefore amplifies the thrombotic consequences of atrial arrhythmia rather than merely coexisting with it.

Yet the same disease state that augments thrombogenicity also impairs primary hemostasis. Platelet function in CKD is frequently abnormal, with reduced adhesion and diminished collagen-induced aggregation independent of dialysis exposure [53]. Qualitative defects predominate despite preserved platelet counts. Clinically, this translates into measurable bleeding vulnerability. In a large prospective cohort of high cardiovascular risk patients, CKD was independently associated with a 1.5-fold increase in major bleeding, with risk rising progressively across CKD stages and with albuminuria [69]. Intracranial and extracranial bleeding both increased. CKD thus produces hemostatic instability characterized by concurrent platelet activation and functional impairment.

When oral anticoagulation is introduced into this unstable environment, the therapeutic margin narrows further. In advanced CKD and dialysis cohorts, randomized data demonstrate similar rates of death and major bleeding between direct oral anticoagulants (DOACs) and vitamin K antagonists (VKAs), without consistent evidence of stroke reduction in hemodialysis populations [66]. Meta-analyses incorporating randomized and observational data similarly report comparable risks of ischemic stroke and major bleeding between DOACs and VKAs in ESRD [70]. Current guideline recommendations therefore emphasize individualized decision-making in advanced CKD, reflecting elevated risks on both sides of the equation and limited definitive evidence [71].

Anticoagulation may also influence renal integrity. Warfarin-related nephropathy illustrates how excessive anticoagulation can precipitate glomerular hemorrhage with tubular obstruction by red blood cell casts, particularly in patients with pre-existing CKD [72,73,74]. Observational data associate anticoagulant-related nephropathy with increased mortality [73]. Although first described with warfarin, similar patterns of acute kidney injury with hematuria have been reported during DOAC therapy [75,76]. Proposed mechanisms include glomerular bleeding, altered protease-activated receptor signaling, and heme-mediated tubular toxicity [74,76]. While causal relationships remain debated, these findings underscore that anticoagulation is not biologically neutral within a kidney already characterized by structural fragility.

Overlaying these processes is persistent systemic inflammation. Advanced CKD is marked by elevated IL-1β, IL-6, TNF-α, CRP, and fibrinogen levels that correlate inversely with renal function [77]. These mediators promote tissue factor expression, endothelial activation, and a procoagulant phenotype. Uremic toxins such as indoxyl sulfate and p-cresyl sulfate exacerbate oxidative stress and impair NO bioavailability. Dialysis partially removes selected solutes but does not normalize inflammatory signaling or endothelial dysfunction [77,78]. Renal function often fluctuates in advanced disease, altering drug exposure and accumulation of bioactive mediators. Hemostasis therefore operates within a dynamic inflammatory and metabolic environment rather than a steady state.

Viewed in this context, the thrombohemorrhagic paradox in AF with CKD is not contradictory but internally consistent. Endothelial activation, vWF dysregulation, platelet dysfunction, systemic inflammation, and variable renal clearance converge to increase both thromboembolic and bleeding risk. Stroke and hemorrhage represent parallel clinical expressions of a disrupted thromboinflammatory interface. Anticoagulation is superimposed upon this fragile equilibrium, modifying thrombin generation while potentially amplifying bleeding susceptibility. Static clinical scores alone are unlikely to capture this complexity. Recognition of the shared biological substrate underlying both complications provides a more coherent framework for understanding risk in patients with AF and CKD.

**Figure 3:**
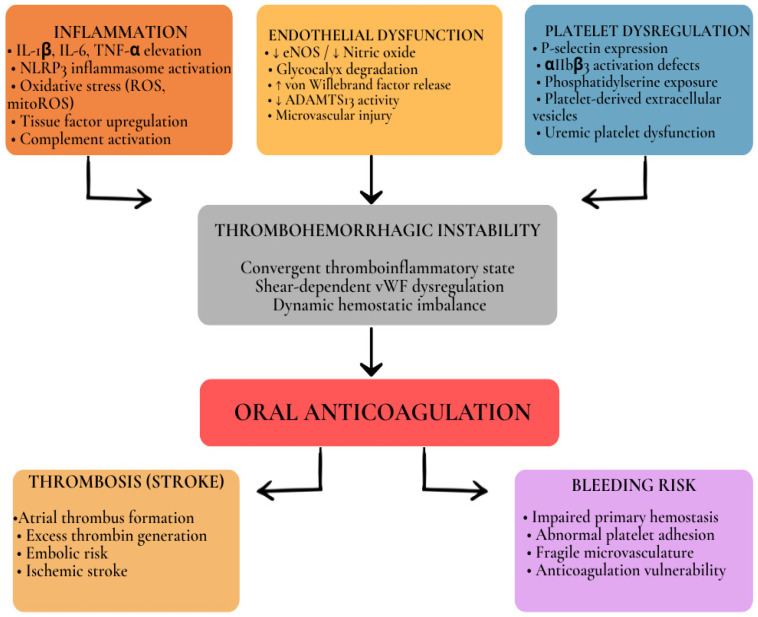
Integrated mechanistic model of thrombohemorrhagic instability in atrial fibrillation with chronic kidney disease. Inflammation, endothelial dysfunction, and platelet dysregulation converge to generate a thromboinflammatory state characterized by excess thrombin generation, shear-dependent von Willebrand factor dysregulation, and qualitative impairment of primary hemostasis. This biological instability predisposes simultaneously to thrombotic complications (including ischemic stroke) and bleeding risk. Oral anticoagulation (vitamin K antagonists or direct oral anticoagulants) intersects both pathways, reducing thrombin-mediated thrombosis while potentially amplifying bleeding vulnerability, particularly in advanced renal dysfunction. Downward arrows (↓) indicate reduced expression, availability, or activity. Upward arrows (↑) indicate increased expression or activity. Solid arrows represent mechanistic contribution or directional influence within the thromboinflammatory cascade.

This shared pathophysiologic substrate can be conceptualized as a disrupted thromboinflammatory interface in which inflammatory activation, endothelial dysfunction, platelet instability, and fluctuating renal clearance interact dynamically rather than independently. Within this integrated framework, thrombosis and bleeding are not opposing phenomena but parallel manifestations of the same biological instability, with anticoagulation superimposed upon an already fragile equilibrium. This integrated mechanistic model is illustrated in Figure 3.

## 8. A Biology-Informed Framework for Anticoagulation in AF with CKD

### 8.1. Limitations of Creatinine Clearance

CrCl, typically estimated using the Cockcroft–Gault equation, remains the cornerstone of direct oral anticoagulant (DOAC) dosing in AF with CKD [9]. This approach reflects the structure of pivotal trials and regulatory approval pathways. It is simple, reproducible, and embedded in guidelines. Yet it was never intended to represent the totality of renal or vascular biology.

CrCl estimates glomerular filtration. It does not measure endothelial injury, inflammatory activation, uremic toxin burden, platelet phenotype, or fibrinolytic capacity. In practice, two patients with similar eGFR values often experience very different trajectories—one remains stable, the other develops bleeding or thromboembolic complications despite guideline-concordant dosing [21]. Filtration alone does not explain that divergence.

Creatinine-based equations are also vulnerable to misclassification. Many patients with AF and CKD are older and sarcopenic. In such individuals, serum creatinine may underestimate the degree of renal impairment. Because DOAC dosing thresholds are fixed to CrCl categories [9], small errors in estimation can translate into meaningful differences in drug exposure. In advanced CKD, where the therapeutic margin is already narrow, this misalignment may be clinically significant.

Albuminuria illustrates another limitation. In a large population-based cohort of older adults with newly diagnosed AF, even modest elevations in UACR were independently associated with higher risks of heart failure, stroke, bleeding, and death at one year [20]. A UACR of 30 mg/g, often considered borderline, was associated with increased hazards of bleeding and mortality compared with minimal albumin excretion. Albuminuria reflects systemic endothelial dysfunction and vascular injury, both central to the thrombohemorrhagic profile of CKD [13,20]. These signals are invisible to CrCl.

Filtration estimates also fail to reflect qualitative differences in retained solutes. Uremic toxins influence endothelial signaling, inflammatory pathways, platelet activation, and coagulation balance [79]. Their accumulation is not linearly proportional to eGFR. Patients within the same CKD stage may therefore differ substantially in inflammatory burden and vascular phenotype. CKD is characterized by concurrent prothrombotic drive and impaired primary hemostasis mediated through endothelial dysfunction, platelet abnormalities, and altered coagulation pathways [13]. A single filtration estimate cannot capture this internal heterogeneity.

CrCl remains operationally necessary. It should not be abandoned. But it should be recognized for what it is: a pharmacokinetic anchor, not a biological risk model.

### 8.2. A Three-Domain Framework for Anticoagulation in AF with CKD

If AF with CKD represents a state of thromboinflammatory instability, anticoagulation decisions should reflect more than a baseline CrCl category. A practical approach can be conceptualized across three interrelated domains: clinical risk, renal trajectory, and biological phenotype.

#### 8.2.1. Domain 1: Clinical Risk

Clinical risk scores form the foundation of anticoagulation decision-making in atrial fibrillation. Current guidelines recommend stroke risk stratification using CHA_2_DS_2_-VASc, with anticoagulation advised in patients exceeding established thresholds. Bleeding risk assessment tools are likewise incorporated into routine practice to support shared decision-making and monitoring [1].

In patients with advanced CKD, however, management becomes more complex. Reviews focused specifically on kidney failure populations emphasize that evidence guiding anticoagulation in stage 4–5 CKD and dialysis is limited, and that treatment strategies require individualized evaluation beyond standard scoring algorithms [8]. Practical guidance addressing AF in CKD similarly highlights uncertainty in advanced renal disease and the need for careful balancing of thromboembolic and hemorrhagic risks [64].

In ESRD, outcome data remain heterogeneous. Meta-analyses evaluating warfarin and direct oral anticoagulants in dialysis populations demonstrate variability in stroke reduction and bleeding risk, with no consistent net benefit across all cohorts [65,66]. These findings indicate that clinical risk scores alone do not fully resolve therapeutic uncertainty in advanced CKD. Accordingly, while CHA_2_DS_2_-VASc and bleeding models define indication and baseline risk, they do not capture the biological heterogeneity that characterizes AF in the setting of CKD.

#### 8.2.2. Domain 2: Renal Trajectory

Renal function is dynamic. In a nationwide cohort of anticoagulated individuals with marginal baseline kidney function, decline in eGFR to <45 mL/min within two years was independently associated with major bleeding, progression to ESRD, and markedly increased mortality [80]. Patients whose renal function remained stable did not demonstrate comparable escalation in risk. The direction and pace of change modify vulnerability during follow-up.

Long-term trajectory analyses reinforce this observation. Among more than 62,000 individuals without baseline AF, distinct eGFR patterns over nearly a decade were associated with differential incidence of new-onset AF; a persistently low trajectory conferred more than a twofold increase in arrhythmic risk compared with a stable high pattern [81]. Sustained renal decline therefore reflects a vascular phenotype predisposed to adverse cardiovascular events.

Serial assessment also provides insight beyond filtration alone. In patients with stage 4–5 CKD followed longitudinally with repeated high-sensitivity cardiac troponin T (hs-cTnT) measurements, time-updated concentrations were strongly associated with mortality, and cumulative exposure further amplified risk [82]. Repeated measurement captures ongoing myocardial stress that a single renal estimate cannot convey.

Multimarker strategies extend this principle. In a cohort undergoing serial evaluation of GDF-15, NT-proBNP, and cardiac troponin I, longitudinal modeling improved prognostic discrimination, and rising biomarker levels preceded adverse clinical events [83]. Although derived in broader cardiovascular populations, the methodological lesson is relevant: biological trajectories may signal instability before overt deterioration.

In non-anticoagulated AF populations, stage 4 CKD independently predicted higher endogenous thrombin potential and prolonged clot lysis time after adjustment for CHA_2_DS_2_-VASc score [84]. These laboratory findings indicate amplified thrombin generation and impaired fibrinolysis within the same renal stage, underscoring that hemostatic phenotype is not uniform across CKD categories.

Prospective data in anticoagulated AF patients with stage 4 CKD further demonstrate biological heterogeneity. In a cohort of 180 individuals, higher concentrations of GDF-15, cystatin C, and hs-cTnT independently predicted major or clinically relevant non-major bleeding, whereas reduced fibrin clot permeability was associated with thromboembolic events [85]. Distinct markers signaled divergent risk domains despite similar eGFR values.

Beyond advanced CKD cohorts, GDF-15 has shown consistent association with bleeding risk. In a community-based population of more than 9000 individuals without prior cardiovascular disease, higher GDF-15 concentrations were linked to incident hospitalization for bleeding [86]. In anticoagulated AF populations, GDF-15 independently predicted major bleeding and was incorporated into the ABC-bleeding score, which outperformed traditional clinical models [87]. In AF with CKD, elevated GDF-15 likely reflects integrated systemic stress—renal dysfunction, inflammation, endothelial perturbation, and myocardial strain—rather than impaired drug clearance alone.

Finally, outcome gradients across CKD stages illustrate how therapeutic margin narrows with advancing renal impairment. In a cohort of 12,714 AF patients stratified by CrCl, three-year rates of net adverse clinical events increased stepwise from early to advanced CKD. The clinical benefit of anticoagulation was evident in stages 1–3 but attenuated in stages 4–5, particularly in patients with lower CHA_2_DS_2_-VASc scores [88]. Renal stage therefore modifies net benefit rather than serving solely as a dosing determinant.

These observations argue against treating CKD stage as biologically homogeneous. Within the same eGFR range, some patients display a thrombin-dominant phenotype, others a bleeding-predominant profile characterized by inflammatory activation and platelet dysfunction. Recognizing that orientation may help refine decisions when the balance between stroke prevention and bleeding risk is uncertain.

### 8.3. Clinical Application: An Adaptive Model

An adaptive approach to anticoagulation in AF with CKD rests on three principles. First, confirm indication. Stroke risk remains the primary driver of anticoagulation. Second, monitor trajectory. Renal decline over time informs both drug exposure and systemic vulnerability [80,81]. Time-updated myocardial injury markers may further clarify evolving risk [82]. Third, recognize phenotype. Biomarkers and selected hemostatic indices may distinguish bleeding susceptibility from thrombotic predominance within the same CKD stage [84,85,86,87].

This framework does not require routine comprehensive biomarker panels for every patient. It supports structured reassessment in those with advanced CKD, fluctuating renal function, prior bleeding, or uncertain net benefit. In such settings, dynamic evaluation may offer greater insight than reliance on a single creatinine threshold.

As kidney function declines, thromboembolic and hemorrhagic risks rise in parallel, and the therapeutic margin narrows [88]. A filtration-based dosing model remains necessary for pharmacokinetic safety, but it does not fully define biological vulnerability. Integrating clinical risk, renal trajectory, and biological phenotype provides a more precise alignment between anticoagulant intensity and the evolving thromboinflammatory state.

The principal clinical and biological signals supporting a precision-oriented approach to anticoagulation in AF with CKD are summarized in Table 3.

## 9. Qualitative Evaluation of the Studies Selected in This Review

Anticoagulation in patients with atrial fibrillation and chronic kidney disease is still largely guided by renal function thresholds derived from pharmacokinetic considerations. Dose adjustment according to CrCl has clear value for drug safety, yet it does not account for the broader biological processes that shape thromboembolic and hemorrhagic risk in this population. The evidence reviewed in this review indicates that vulnerability in AF with CKD emerges from interacting mechanisms—persistent inflammation, endothelial activation, myocardial injury, altered coagulation, and progressive renal decline. A single creatinine-based estimate cannot adequately reflect this evolving landscape.

In clinical practice, several implications follow. Renal function should be interpreted longitudinally rather than as an isolated measurement. A sustained downward trend over months may signal rising bleeding and mortality risk even when absolute values remain within an established dosing category. Serial assessment becomes particularly relevant in older or frail individuals, in whom small changes in renal function may translate into meaningful shifts in drug exposure.

Circulating biomarkers add another dimension. Markers of myocardial injury and systemic stress may help identify patients in whom competing mortality risk or bleeding susceptibility begins to outweigh anticipated thromboembolic benefit. In selected cases, particularly when clinical equipoise exists, such information may inform closer follow-up, reassessment of concomitant therapies, or reconsideration of anticoagulation intensity.

Advanced CKD should not be viewed as a uniform stage. Within the same eGFR range, substantial heterogeneity exists in thrombin-generating capacity, fibrinolytic balance, endothelial activation, and biomarker profiles. Two patients with similar filtration rates may therefore face very different biological risks. Recognizing this heterogeneity supports individualized discussion rather than automatic escalation or withdrawal of anticoagulation solely on the basis of stage classification.

Precision anticoagulation, as proposed here, does not imply indiscriminate laboratory testing. It suggests a structured model of reassessment: periodic evaluation of renal trajectory, selective incorporation of validated biomarkers when uncertainty arises, and explicit consideration of net clinical benefit in advanced disease. This approach may be especially relevant in stage 4 CKD, where event rates are high and the therapeutic margin between stroke prevention and bleeding narrows considerably.

Several research directions deserve priority. Prospective studies are needed to determine whether biomarker-informed anticoagulation strategies improve outcomes compared with standard algorithms based exclusively on renal function and clinical scores. Trials that incorporate time-updated renal function and serial biomarker measurements into adaptive treatment strategies would clarify whether dynamic risk assessment can reduce major bleeding without compromising stroke prevention. The interaction between inflammatory burden, endothelial dysfunction, and anticoagulant pharmacodynamics in advanced CKD also warrants further translational investigation.

Future models should avoid reliance on a single biomarker. Panels that integrate renal trajectory, myocardial injury, systemic stress, and hemostatic balance are more likely to approximate the underlying biological state. At the same time, feasibility must remain central. Risk stratification tools must be practical, reproducible, and applicable within routine clinical workflows if they are to influence real-world decision-making.

For patients with AF and CKD, anticoagulation decisions rarely present as simple yes-or-no choices. They unfold along a continuum of evolving risk. CrCl remains an important anchor, but it should be interpreted within a broader clinical and biological framework. The task ahead is to translate mechanistic insight and biomarker evidence into pragmatic strategies that refine existing practice while preserving its simplicity and safety.

## 10. Conclusions

Atrial fibrillation and chronic kidney disease intersect along a shared biological axis defined by inflammation, endothelial dysfunction, platelet dysregulation, and altered coagulation. This interaction generates a state of hemostatic instability in which thromboembolic and hemorrhagic events arise from common mechanistic roots rather than opposing pathways. Endothelial activation with increased Vwf release, impaired ADAMTS13-mediated regulation, oxidative stress-driven NO depletion, and sustained cytokine signaling converge with uremic platelet dysfunction and enhanced thrombin generation. The result is a disrupted vascular interface that predisposes simultaneously to stroke and bleeding.

In this context, anticoagulation is superimposed upon an already unstable equilibrium. CrCl-based dosing remains a practical anchor, yet it reflects only one dimension of a multidimensional disease process. Renal filtration does not capture inflammatory burden, endothelial injury, myocardial stress, platelet exhaustion, or dynamic shifts in coagulation potential. Clinical observation confirms this biological complexity: as kidney function declines, thrombotic and hemorrhagic risks increase in parallel, and the therapeutic margin narrows, particularly in advanced CKD and dialysis populations.

Reframing anticoagulation risk in AF with CKD therefore requires moving beyond a static filtration model toward a biology-informed framework. Renal trajectory, circulating markers of myocardial injury and systemic stress, and indices of hemostatic phenotype provide complementary insight into evolving vulnerability. Such parameters do not replace established clinical scores but refine their interpretation, particularly when decisions are finely balanced. Within this integrated view, thrombosis and bleeding represent different clinical expressions of the same disrupted thromboinflammatory substrate.

The challenge ahead lies in translating mechanistic understanding into clinically applicable strategies. Prospective validation of dynamic risk models, incorporation of time-updated renal assessment, and selective use of biomarker-guided stratification may help better align thromboembolic protection with hemorrhagic safety. For patients living with both AF and CKD, anticoagulation decisions unfold within a continuum of biological change. Recognizing and responding to that dynamic complexity offers a path toward more individualized and physiologically grounded care.

## Figures and Tables

**Table 1:** Key thromboinflammatory mediators and pathways linking inflammation to coagulation activation and propagation.

Mediator/Pathway	Trigger/Cellular Source	Procoagulant Effect	Thromboinflammatory Integration
Tissue factor (TF) induction by inflammatory signaling [23,33,36]	DAMPs; monocyte/macrophage activation; HMGB1; type I interferon signaling; hypoxia (PKC–Egr-1; HIF-1α pathways)	Upregulation of TF expression and activity; TF–FVIIa complex initiates FX activation and thrombin generation	Positions TF as a central convergence point between innate immune activation and coagulation initiation
TF-positive extracellular vesicles (EVs) [34,35]	Inflammatory activation of monocytes and vascular cells; EV release in inflammatory states	Dissemination of TF activity; expansion of procoagulant surfaces supporting thrombin generation	Extends coagulation potential beyond the site of injury; links cellular inflammation to systemic thrombogenicity
Platelet-derived extracellular vesicles (pEVs) [36]	Activated platelets (triggered by thrombin, collagen, ADP, Ca^2+^ influx)	Exposure of phosphatidylserine; carriage of TF, FVa, and FVIII; support of tenase and prothrombinase complex assembly	Transport inflammatory mediators and complement components; amplify platelet–leukocyte–endothelial interactions
Phosphatidylserine (PS) exposure [33,36]	Activated platelets and pEVs; NET-associated procoagulant surfaces	Provides catalytic platform for tenase and prothrombinase complexes, enhancing thrombin generation	Integrates platelet activation and NET-associated surfaces into propagation of intravascular coagulation
Protein disulfide isomerase (PDI)-mediated TF “decryption” [36,37,38,39,40,41,42,43,44,45]	Released from activated platelets and endothelial cells	Enhances TF procoagulant activity by promoting conformational activation (“decryption”)	Links cellular activation to increased TF functionality within thromboinflammatory settings
Thrombin feedback amplification (FV, FVIII, FXI activation) [33,34,36]	Propagation phase following TF-mediated initiation	Thrombin amplifies its own generation via activation of FV, FVIII, and FXI; stabilizes fibrin formation	Establishes feed-forward coupling between inflammatory initiation and sustained coagulation propagation
Thrombin–protease-activated receptor (PAR) signaling [33,34,36]	Thrombin activation of PAR-1 (predominant) and PAR-4 on platelets and endothelium	Enhances platelet activation, integrin signaling, granule release, and endothelial adhesion molecule expression	Couples coagulation protease activity to endothelial activation and cytokine signaling
Neutrophil extracellular traps (NETs) [22,34,35,38]	Neutrophil activation and NETosis	Provide scaffold for platelet adhesion and fibrin deposition; histones and cfDNA enhance thrombin generation	Bridge innate immune effector mechanisms with intravascular coagulation; support thrombus stability
Contact pathway activation (FXII → FXI) [34,36]	Extracellular DNA and negatively charged NET components	FXII activation triggers intrinsic pathway amplification and additional thrombin generation	NET-associated DNA links immune activation to intrinsic pathway propagation
TF pathway reinforcement via TFPI cleavage [36]	Neutrophil-derived proteases (e.g., elastase) within thromboinflammatory milieu	Cleavage of TF pathway inhibitor (TFPI) reduces anticoagulant restraint, enhancing TF-driven coagulation	Connects neutrophil activation and NET-associated proteolysis to loss of anticoagulant control
Complement–coagulation cross-talk [33,34]	Complement activation via PAMPs/DAMPs during inflammation	Complement components enhance TF expression, endothelial activation, and platelet reactivity; coagulation proteases reciprocally activate complement pathways	Integrates complement activation with platelet, endothelial, and coagulation signaling within thromboinflammation
C3a and C5a-mediated vascular activation [34]	Complement activation products (C3a, C5a)	Promote endothelial activation and procoagulant responses, including TF upregulation	Reinforce endothelial–leukocyte interactions and NET formation within thromboinflammatory loops
Innate immune receptor (TLR)-driven thromboinflammation [37]	TLR recognition of PAMPs/DAMPs on immune and vascular cells	Induces cytokine release, platelet activation, and endothelial TF expression	Positions innate immune sensing as an upstream driver of coagulation activation and NET-mediated thrombosis
Endothelial anticoagulant pathway suppression (Protein C/thrombomodulin axis) [33,34]	Inflammatory cytokines; endothelial activation	Downregulation of thrombomodulin and impaired protein C activation; reduced anticoagulant and anti-inflammatory signaling	Loss of endogenous anticoagulant restraint contributes to sustained thrombin generation
Inflammation-associated hypofibrinolysis (PAI-1 upregulation) [33,34]	Cytokine-driven endothelial activation	Increased plasminogen activator inhibitor-1 (PAI-1); impaired fibrinolysis and enhanced clot persistence	Favors thrombus stability within chronic inflammatory states such as AF and CKD

Abbreviations: ADP, adenosine diphosphate; AF, atrial fibrillation; Ca^2+^, calcium ion; cfDNA, cell-free DNA; C3a, complement component 3a; C5a, complement component 5a; CKD, chronic kidney disease; DAMPs, damage-associated molecular patterns; EVs, extracellular vesicles; FX, coagulation factor X; FVa, activated coagulation factor V; FVIIa, activated coagulation factor VII; FVIII, coagulation factor VIII; FXI, coagulation factor XI; FXII, coagulation factor XII; HIF-1α, hypoxia-inducible factor-1 alpha; HMGB1, high mobility group box 1; NETs, neutrophil extracellular traps; PAI-1, plasminogen activator inhibitor-1; PAMPs, pathogen-associated molecular patterns; PAR, protease-activated receptor; PAR-1, protease-activated receptor-1; PAR-4, protease-activated receptor-4; PDI, protein disulfide isomerase; pEVs, platelet-derived extracellular vesicles; PKC, protein kinase C; PS, phosphatidylserine; TF, tissue factor; TFPI, tissue factor pathway inhibitor; TLR, Toll-like receptor.

**Table 2:** Determinants of Hemorrhagic Risk in CKD and AF: Mechanisms of Platelet Activation and Dysfunction.

Determinant	Primary Trigger	Pathophysiological Mechanism	Hemostatic Consequence	Clinical Expression
Anemia (↓ Hematocrit) [57,58]	Reduced RBC mass	↓ Platelet margination under shear; impaired platelet–wall interaction; reduced rheological support for thrombin generation	Impaired primary hemostasis; smaller platelet plug formation	Increased bleeding risk, particularly under antithrombotic therapy
Hemodialysis Membrane Contact [60,61]	Blood–artificial surface interaction	Protein adsorption (fibrinogen, FXII) → intrinsic pathway activation; platelet adhesion via αIIbβ3; thrombin generation despite heparin	Repetitive platelet activation and microthrombus formation	Platelet consumption; intradialytic activation
Complement Activation during HD [60,61,62]	Activation of classical, lectin, and alternative pathways	C3a/C5a generation; terminal complement complex formation; complement–coagulation amplification loop	Inflammatory amplification of coagulation; endothelial activation	Unstable hemostatic equilibrium; progressive platelet dysfunction
Repetitive Dialysis Exposure [61,62]	Chronic extracorporeal circulation	Recurrent platelet activation; phosphatidylserine exposure; granule depletion	Functional platelet attenuation (“exhaustion”)	Bleeding susceptibility despite activation markers
Elevated vWF in ESRD [63]	Endothelial activation; reduced renal clearance	Increased vWF antigen and activity; altered multimer composition	Dysregulated platelet tethering and aggregation	Associated with gastrointestinal bleeding risk
vWF–ADAMTS13 Imbalance [46]	↓ ADAMTS13 activity with ↑ vWF levels	Reduced cleavage of ultra-large vWF multimers; persistence of highly adhesive multimers	Enhanced shear-dependent thrombogenicity with reduced multimer regulation	Increased mortality risk; coexistence of thrombotic and bleeding events

Abbreviations: AF, atrial fibrillation; ADAMTS13, A Disintegrin and Metalloproteinase with Thrombospondin Type 1 Motif Member 13; CKD, chronic kidney disease; C3a, complement component 3a; C5a, complement component 5a; ESRD, end-stage renal disease; FXII, coagulation factor XII; HD, hemodialysis; RBC, red blood cell; vWF, von Willebrand factor; αIIbβ3, platelet integrin glycoprotein IIb/IIIa. Symbols: ↓ indicates decreased levels or activity; ↑ indicates increased levels or activity.

**Table 3:** Clinical and Biological Signals Supporting Precision Anticoagulation in Atrial Fibrillation with Chronic Kidney Disease.

Signal	Biological Domain	Clinical Outcomes Associated	Quantitative Signal	Precision Implication
Renal trajectory (eGFR decline over time) [80,81]	Dynamic renal reserve; evolving drug exposure	Major bleeding, ESRD, mortality; incident AF	eGFR decline to <45 mL/min within 2 years associated with ↑ bleeding (aHR~1.5) and markedly ↑ mortality (aHR > 9); low-stable trajectory HR~2.3 for incident AF	Renal function should be interpreted longitudinally; dosing and monitoring intensity should reflect trajectory rather than a single baseline estimate.
High-sensitivity cardiac troponin T (serial measurement) [82]	Ongoing myocardial injury	Mortality	Per SD increase HR~3.3 for death	Serial myocardial injury assessment identifies evolving vulnerability not captured by CrCl alone.
GDF-15 [85,86,87]	Systemic stress; bleeding susceptibility	Bleeding hospitalization; major/CRNM bleeding	Highest vs lowest quartile HR~2.0 for bleeding; independently predictive in stage 4 CKD AF	Identifies biological bleeding risk beyond renal function; supports biomarker-augmented bleeding stratification.
Cystatin C [85]	Renal dysfunction beyond creatinine; frailty signal	Major/CRNM bleeding; mortality	Strong independent association with bleeding (HR > 9 in stage 4 CKD cohort)	May refine risk classification in advanced CKD where creatinine-based estimates are unstable.
Hemostatic phenotype markers (ETP, CLT, fibrin clot permeability) [64,85]	Thrombin generation; fibrinolytic resistance; clot structure	Thromboembolism and laboratory prothrombotic profile	Stage 4 CKD predicted high ETP (OR~9) and prolonged CLT (OR~3.6); reduced clot permeability associated with TE	Demonstrates biological heterogeneity within CKD stages; thrombotic and bleeding risk are not uniformly expressed across similar eGFR categories.
Multimarker serial strategy (GDF-15 + NT-proBNP + cTnI) [83]	Integrated dynamic profiling	Composite adverse outcomes	Combined panel AUC~0.79	Repeated multimarker assessment may outperform single-point evaluation; methodological foundation for precision models.
Net clinical event gradient across CKD stages [82]	Stage-dependent therapeutic margin	Net adverse clinical events	Progressive rise in 3-year NACE; anticoagulation benefit attenuated in stages 4–5	Advanced CKD requires individualized risk enrichment rather than uniform escalation of anticoagulation intensity.

Abbreviations: AF, atrial fibrillation; aHR, adjusted hazard ratio; AUC, area under the curve; CKD, chronic kidney disease; CLT, clot lysis time; CRNM, clinically relevant non-major; cTnI, cardiac troponin I; eGFR, estimated glomerular filtration rate; ETP, endogenous thrombin potential; ESRD, end-stage renal disease; GDF-15, growth differentiation factor-15; HR, hazard ratio; CrCl, creatinine clearance: NACE, net adverse clinical events; NT-proBNP, N-terminal pro–B-type natriuretic peptide; OR, odds ratio; SD, standard deviation; TE, thromboembolism.

## Data Availability

No new data were created or analyzed in this study. Data sharing is not applicable to this article.

## Abbreviations

The following abbreviations are used in this manuscript:

AF	Atrial fibrillation
ADMA	Asymmetric dimethylarginine
ADP	Adenosine diphosphate
ATP	Adenosine Triphosphate
BH2	Dihydrobiopterin
BH4	Tetrahydrobiopterin
Ca^2+^	Calcium Ion
CaMKII	Ca^2+^/Calmodulin-Dependent Protein Kinase II
C3a	Complement component 3a
C5a	Complement component 5a
CKD	Chronic kidney disease
CrCl	Creatinine clearance
CRP	C-Reactive Protein
ESRD	End-stage renal disease
eGFR	Estimated glomerular filtration rate
eNOS	Endothelial nitric oxide synthase
IL-1β	Interleukin-1 Beta
IL-6	Interleukin-6
IL-18	Interleukin-18
mitoROS	Mitochondrial reactive oxygen species
NADPH	Nicotinamide adenine dinucleotide phosphate
NETs	Neutrophil extracellular traps
NLRP3	NOD-Like Receptor Family Pyrin Domain Containing 3
NO	Nitric oxide
PAR	Protease-activated receptor
PEVs	Platelet-derived extracellular vesicles
RAAS	Renin–Angiotensin–Aldosterone System
ROS	Reactive Oxygen Species
RyR2	Ryanodine Receptor 2
SDMA	Symmetric dimethylarginine
SERCA	Sarcoplasmic Reticulum Ca^2+^-ATPase
TF	Tissue factor
TNF-α	Tumor Necrosis Factor Alpha
UACR	Urinary Albumin-to-Creatinine Ratio
vWF	von Willebrand factor
